# Protein quality control of *N*-methyl-D-aspartate receptors

**DOI:** 10.3389/fncel.2022.907560

**Published:** 2022-07-22

**Authors:** Taylor M. Benske, Ting-Wei Mu, Ya-Juan Wang

**Affiliations:** Department of Physiology and Biophysics, School of Medicine, Case Western Reserve University, Cleveland, OH, United States

**Keywords:** NMDA receptors, variants, proteostasis, endoplasmic reticulum, surface, folding

## Abstract

N-methyl-D-aspartate receptors (NMDARs) are glutamate-gated cation channels that mediate excitatory neurotransmission and are critical for synaptic development and plasticity in the mammalian central nervous system (CNS). Functional NMDARs typically form *via* the heterotetrameric assembly of GluN1 and GluN2 subunits. Variants within *GRIN* genes are implicated in various neurodevelopmental and neuropsychiatric disorders. Due to the significance of NMDAR subunit composition for regional and developmental signaling at synapses, properly folded receptors must reach the plasma membrane for their function. This review focuses on the protein quality control of NMDARs. Specifically, we review the quality control mechanisms that ensure receptors are correctly folded and assembled within the endoplasmic reticulum (ER) and trafficked to the plasma membrane. Further, we discuss disease-associated variants that have shown disrupted NMDAR surface expression and function. Finally, we discuss potential targeted pharmacological and therapeutic approaches to ameliorate disease phenotypes by enhancing the expression and surface trafficking of subunits harboring disease-associated variants, thereby increasing their incorporation into functional receptors.

## Introduction

Ionotropic glutamate receptors (iGluRs) are a family of tetrameric glutamate-gated cation channels. They mediate excitatory synaptic transmission in the CNS of vertebrates. There are three major subtypes of iGluRs: α-amino-3-hydroxy-5-methyl-4-isoxazolepropionic acid receptors (AMPARs), kainate receptors, and *N*-methyl-D-aspartate receptors (NMDARs). NMDARs mediate the slow component of synaptic current in excitatory signaling and play a critical role in the formation and maturation of excitatory synapses. Thereby, NMDARs have been implicated in learning, memory, synaptic plasticity, and long-term potentiation (Hansen et al., 2021). The *GRIN* gene family encodes the GluN subunits that combine to form functional receptors. The obligatory GluN1 subunits bind glycine and are encoded by a single gene, *GRIN1*, which has eight splice variants. NMDARs form heterotetramers, most commonly composed of two GluN1 subunits which can combine with two additional subunits; either the glutamate-binding subunits, which can be GluN2(A-D), or less commonly, the glycine-binding GluN3(A-B) subunits, each encoded by a unique gene (*GRIN2A*, *GRIN2B*, *GRIN3A*, etc.) (Traynelis et al., 1995, 2010). NMDARs are most commonly composed of GluN1 and GluN2 subunits, particularly GluN2A and GluN2B (Monyer et al., 1994; Paoletti et al., 2013; Lee et al., 2014). NMDARs containing GluN2A subunits exhibit three-fold faster decay times and reduced affinity for glutamate than GluN2B-containing receptors (Stern et al., 1992; Erreger et al., 2007; Wyllie et al., 2013). The GluN3 subunits can form non-conventional NMDARs that exhibit atypical biophysical properties including decreased permeability to Ca^2 +^ and insensitivity to voltage-dependent Mg^2+^ block of the channel. GluN3-containing receptors have important functions that counteract the role of conventional NMDARs such as delaying synapse maturation and destabilizing the synapse to promote dendritic pruning (Sasaki et al., 2002; Grand et al., 2018). Readers are directed to the following reviews: Pérez-Otaño et al. (2016) and Crawley et al. (2022), for a more detailed examination of GluN3 subunits, as they are beyond the scope of the current review.

*N*-methyl-D-aspartate receptors within the CNS have been found to consist primarily of triheteromeric receptors composed of GluN1/GluN2A/GluN2B subunits (Chazot et al., 1994; Sheng et al., 1994; Chazot and Stephenson, 1997b; Luo et al., 1997; Rauner and Köhr, 2011). Other triheteromeric NMDARs, including GluN1/GluN2A/GluN2D and GluN1/GluN2B/GluN2D, have been observed in the human spinal cord as well as the rat thalamus and midbrain (Sundström et al., 1997; Dunah et al., 1998). Many research studies to date have focused on diheteromeric NMDARs containing identical GluN2 or GluN3 subunits. However, triheteromeric NMDARs containing a combination of GluN2 and/or GluN3 subunits have distinct channel gating kinetics and pharmacology from diheteromeric receptors (Paoletti et al., 2013; Stroebel et al., 2018).

**FIGURE 1:**
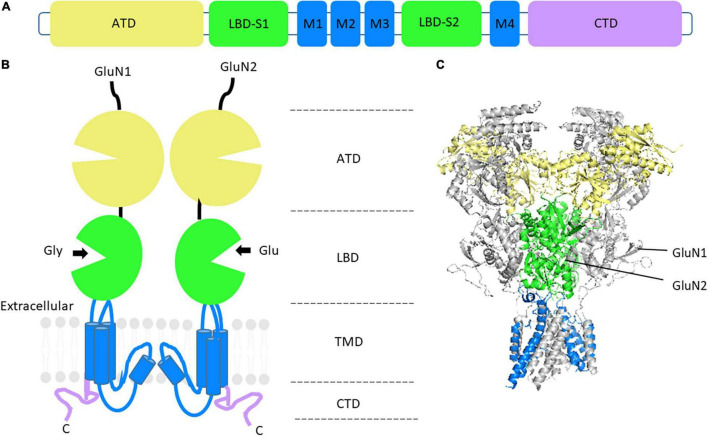
NMDAR structure and GluN subunit domain architecture. **(A)** Linear representation of GluN subunit architecture. **(B)** Topology of a GluN1 and GluN2 subunit dimer. The extracellular domain consists of the amino-terminal domain (ATD) in yellow and the ligand-binding domain (LBD) in green. The transmembrane domains (TMD) and associated linker regions are shown in blue and the intracellular carboxy-terminal domain (CTD) is in purple. **(C)** Side view of the crystal structure of the rat GluN1_GluN2B heterotetrameric NMDAR, without CTD. GluN1 subunits are shown in gray. PDB: 4PE5 (Karakas and Furukawa, 2014).

All GluN subunits share the same domain architecture (Figure 1) consisting of a large amino-terminal domain (ATD), a ligand-binding domain (LBD) comprised of two segments, S1 and S2, and a transmembrane domain (TMD), consisting of three transmembrane helices and one reentrant loop, forming the pore, and an intracellular, intrinsically disordered, C-terminal domain (CTD) (Petralia et al., 2009; Karakas and Furukawa, 2014). NMDARs are unique in the iGluR family in that they have a voltage-dependent Mg^2+^ block, high Ca^2+^ permeability, and their activation requires binding of endogenous co-ligands, glycine (or D-serine) in addition to glutamate, resulting in conformational changes that open the channel (Kampa et al., 2004). Depolarization of the postsynaptic neuron relieves the voltage-dependent Mg^2+^ block allowing Ca^2+^ and other cations to permeate through the receptor (Johnson and Ascher, 1987). Once Ca^2+^ has entered the postsynaptic cell, it can act as a second messenger to regulate gene expression, post-translational modifications, and modify synaptic strength (Lynch et al., 1983; Watanabe et al., 2002; Kawamoto et al., 2012).

### Disease-associated variants of *N*-methyl-D-aspartate receptors

Disease-associated variants (DAVs) within NMDAR subunits lead to dysregulated signaling and distinct disease pathologies. NMDARs are implicated in a variety of neurodevelopmental (Burnashev and Szepetowski, 2015) and neuropsychiatric disorders, including autism spectrum disorder, intellectual disabilities, epilepsy, attention-deficit/hyperactivity disorder, Alzheimer’s disease, and schizophrenia (Goff and Coyle, 2001; Jensen et al., 2009; Endele et al., 2010; Tarabeux et al., 2011; Lemke et al., 2014; Soto et al., 2014; Burnashev and Szepetowski, 2015; Lee et al., 2015; Qin et al., 2016; Liu et al., 2019).

Many genes that have been linked to neurodevelopmental disorders are highly intolerant to mutations, indicating that variants are more likely to result in disease states and cause disorders compared to tolerant genes (Coe et al., 2019). *GRIN* genes are no exception, as an analysis of the genetic variation within the healthy population using residual variation intolerance scores (RVIS) showed fewer single nucleotide polymorphisms present than anticipated. The *GRIN* RVISs indicate that *GRIN2A* is among the 3.89% of most intolerant human genes while *GRIN2B* is among the top 1.07% of most intolerant genes. Interestingly, these *GRIN2* genes are estimated to be more intolerant to variation than the obligatory *GRIN1* which is among the top 6.72% of most intolerant human genes. The whole tolerance list of human genes can be found at https://genic-intolerance.org/data/GenicIntolerance_v3_12Mar16.txt. While they do not always contribute to disease states, the most common genetic variation amongst people are single-nucleotide polymorphisms (Petrovski et al., 2013). *GRIN* variant intolerance is further supported by the probability of being loss-of-function intolerant scores (pLI) and the observed/expected (o/e) ratios of *GRIN* genes obtained through the Genome Aggregation Database (gnomAD), a collection of genome sequencing data (Karczewski et al., 2020). pLI scores close to 1 and o/e ratios close to zero indicate that a gene is most intolerant to variation (Lek et al., 2016). These scores correlate well to the RVISs in that both *GRIN2A* and *GRIN2B* both have a pLI of 1, and an o/e of 0.08 and 0, respectively. *GRIN1* (pLI = 0.98; o/e = 0.17) and *GRIN2D* (pLI = 1; o/e = 0.05) are also intolerant to variations while *GRIN2C* (pLI = 0; o/e = 0.48) is more tolerant to variation compared to other *GRIN* genes. Together these scores indicate that *GRIN* genes are extremely intolerant to mutations, and thereby variants are likely to be associated with disease states. Still, over 1,000 missense mutations are reported in ClinVar across all *GRIN* genes; of these 20% have been classified as pathogenic or likely pathogenic (accessed 2/19/2022). Further, a database, GRINdb, compiled available *GRIN* mutants reported across ClinVar, LOVD, gnomAD, Uniprot, and other reference groups such as the CFERV, and has reported over 4,000 non-redundant missense mutations across all *GRIN* genes (García-Recio et al., 2021).

Variants are found in all GluN subunits but are not distributed evenly across subunits or domains and are most prevalent in the GluN2 subunits, especially those that are more commonly expressed, GluN2A and GluN2B. Many of these mutations are enriched in the LBD and the TMD regions, while fewer have been identified within the ATD and CTD (XiangWei et al., 2018). Whether the roles the ATD and CTD play in the biogenesis and trafficking of NMDARs influences the number of variants observed within these subdomains has not been determined. Several reviews are available that summarize the known roles of these regions (Horak et al., 2014, 2021; Ishchenko et al., 2021; Warnet et al., 2021). Further, a detailed review summarizing the impacts of DAVs in specific domain regions of the receptor is presented by Amin et al. (2021b). The uneven distribution of variants may indicate certain residues, in which variants are not observed, have not yet been identified in patient genomic screening, or are essential in the function or trafficking of the receptor. Certainly, DAVs are generally observed less in the population due to selective pressures. Additionally, variants that are unable to produce a functional receptor are likely to be embryonically or perinatally lethal (Sprengel et al., 1998).

Here, we focus on missense mutations within the common subunits, GluN1, GluN2A, and GluN2B, which result in reduced surface expression. Table 1 summarizes the clinical phenotypes and the functional consequences of such variants. Variants in which characterization has shown reduced currents but whose surface expression was not quantified are not considered in this review. While it is probable that these variants have some magnitude of disrupted trafficking and/or surface expression, other variables could contribute to reduced current amplitude, independent of surface expression, such as changes to agonist affinity, open probability of the channel, and Mg^2+^ sensitivity (XiangWei et al., 2018). The GluN2 subunits display unique structural characteristics resulting in differing biophysical properties such as kinetics, mobility, signal transduction, and responses to pharmacological treatments, thus leading to distinct pathologies when dysregulated (Paoletti et al., 2013; Rajani et al., 2020). GluN2A variants are commonly associated with epilepsy and intellectual disorders while GluN2B variants are more commonly associated with neurodevelopmental and intellectual disorders (Myers et al., 2019). This is likely a key factor in their differential expression during development. GluN2B subunits are predominant during prenatal development at nascent synapses and immature neurons and exhibit an age-dependent decrease (Watanabe et al., 1992, 1993; Monyer et al., 1994; Jantzie et al., 2015). GluN2A subunits replace GluN2B subunits during postnatal development as neurons mature and the amount of GluN2A-containing receptors increases into adulthood (Akazawa et al., 1994).

**TABLE 1:** Disease-associated variants with reduced surface expression* within GluN1, GluN2A, and GluN2B subunits.

Subunit	Variant	Subdomain	Functional defect	Disease phenotype	Functional consequence	References
GluN1	P532H	LBD-S1	ND	Epi	↓ Glu potency, ↓ expression, ↓ current density, ↑ Zn inhibition, ↓ Popen	Zhang J. et al., 2021
GluN1	D552E	S1-M1	LOF	Epi, ID	↓ Glu and Gly potency, ↓ expression, ↓ current density	Ogden et al., 2017
GluN1	P557R	S1-M1	LOF	ID	↑ Glu and Gly potency, ↓ expression, ↓ current density	Ogden et al., 2017
GluN1	G618R	M2	LOF	ID, Hpt	↓ Expression, non-functional	Li et al., 2019
GluN1	G620R	M2	LOF	ID	↓ Glu and Gly potency, ↓ expression, ↓ current density, ↓ Mg block	Chen et al., 2017; Fry et al., 2018
GluN1	M641I	M3	ND	Epi, ID	↓ Expression	Lemke et al., 2016; Kolcheva et al., 2021
GluN1	A645S	M3	ND	Epi, ID, CVI	↓ Expression	Lemke et al., 2016; Kolcheva et al., 2021
GluN1	Y647S	M3	LOF	Epi, ID, IS	↓ Current amplitude	Lemke et al., 2016; Kolcheva et al., 2021
GluN1	S688Y	LBD-S2	ND	ID	↓ Surface expression GluN3A receptor	Zehavi et al., 2017; Skrenkova et al., 2020
GluN1	D789N	LBD-S2	ND	Epi, ID	↓ Current, ↓ expression	Fry et al., 2018
GluN2A	P79R	ATD	LOF	Epi, ADHD	↓ Glu and Gly potency, ↓ expression	Lemke et al., 2013; Addis et al., 2017
GluN2A	I184S	ATD	LOF	Epi, ID	↓ Current, ↑ activation and deactivation time, ↓ expression	Sibarov et al., 2017
GluN2A	C231Y	ATD	LOF	Epi, ID, LKS	↓ Glu and Gly potency, ↓ current, ↓ expression	Lemke et al., 2013; Addis et al., 2017
GluN2A	C436R	LBD-S1	LOF	Epi	↑ Glu potency, ↓ Gly potency, ↓ current density, ↓ charge transfer, ↓ expression	Lemke et al., 2013; Swanger et al., 2016; Addis et al., 2017
GluN2A	G483R	LBD-S1	LOF	Epi, ID	↓ Glu potency, ↓ deactivation delay time, ↓ current density, ↓ charge transfer, ↓ expression	Lesca et al., 2013; Swanger et al., 2016; Addis et al., 2017
GluN2A	R504W	LBD-S1	LOF	Epi	↑ Deactivation delay time, ↓ expression	Lesca et al., 2013; Swanger et al., 2016
GluN2A	R518H	LBD-S1	LOF	Epi, ID, ASD	↓ Current density, ↓ expression, ↑ activation and deactivation time	Swanger et al., 2016
GluN2A	T531M	LBD-S1	LOF	Epi, ID	↓ Current density, ↓ expression	Carvill et al., 2013; Swanger et al., 2016
GluN2A	V685G	LBD-S2	LOF	Epi, LKS	↓ Glu potency, ↓ charge transfer, ↓ current density, ↓ deactivation decay time↓ expression,	Swanger et al., 2016
GluN2A	I694T	LBD-S2	LOF	Epi, LKS	↓ Glu potency, ↓ Popen, ↓charge transfer, ↓expression	Swanger et al., 2016
GluN2A	P699S	LBD-S2	LOF	Epi	↑ Glu potency, ↓ Popen, ↓charge transfer, ↓expression	Lemke et al., 2013; Swanger et al., 2016
GluN2A	M705V	LBD-S2	LOF	Epi, ID	↓ Glu potency, ↓ Popen, ↓charge transfer, ↓expression	Lemke et al., 2013; Swanger et al., 2016; Addis et al., 2017
GluN2A	E714K	LBD-S2	LOF	Epi, ID, ADHD	↓ Expression	Lemke et al., 2013; Swanger et al., 2016; Addis et al., 2017
GluN2A	A716T	LBD-S2	LOF	Epi, verbal dyspraxia	↓ Glu potency, ↓ deactivation decay time, ↓ expression	Lesca et al., 2013; Swanger et al., 2016
GluN2A	A727T	LBD-S2	LOF	Epi, ID	↓ Glu potency, ↓ Popen, ↓ charge transfer, ↓ expression	Lemke et al., 2013; Swanger et al., 2016
GluN2A	D731N	LBD-S2	LOF	Epi, verbal dyspraxia, DD	↓ Glu and Gly potency, ↑ H^+^/Zn inhibition, ↓ Popen, ↓ charge transfer, ↓ expression	Swanger et al., 2016; Gao et al., 2017
GluN2A	K772E	LBD-S2	LOF	Epi	↓ Glu potency, ↓ current density, ↓ Popen, ↓ charge transfer, ↓ expression	Lemke et al., 2013; Swanger et al., 2016
GluN2B	E413G	LBD-S1	LOF	ID, Hpt	↓ Glu potency, ↓ deactivation decay time, ↓ current density, ↓ charge transfer, ↓ expression	Swanger et al., 2016; Platzer et al., 2017; Wells et al., 2018
GluN2B	C436R	LBD-S1	LOF	Epi, ID	↓ Current density, ↓ expression	Swanger et al., 2016; Platzer et al., 2017
GluN2B	C456Y	LBD-S1	LOF	ASD, ID	↑ Glu potency, ↓ Gly potency, ↓ charge transfer, ↓ current density, ↓ expression	Swanger et al., 2016
GluN2B	C461F	LBD-S1	LOF	Epi, ID, LGS	↓ Glu and Gly potency, ↓ deactivation decay time, ↓ current density,↓ charge transfer, ↓ expression	Allen et al., 2013; Swanger et al., 2016; Platzer et al., 2017
GluN2B	R540H	LBD-S1	GOF	Epi, ID	↑ Glu and Gly potency, ↑ deactivation decay time, ↓ expression, ↓ Mg inhibition, ↑ calcium permeability	Lemke et al., 2014; Swanger et al., 2016; Platzer et al., 2017
GluN2B	P553L	S1-M1	LOF	ID, Hpt, dysmorphic	↓ Current density, Glu insensitivity, ↓ expression	de Ligt et al., 2012; Vyklicky et al., 2018
GluN2B	W607C	M2	ND	ID, dysmorphic	↓ Current density,↓ expression, ↓ Popen, ↓ Glu potency	Yavarna et al., 2015; Vyklicky et al., 2018
GluN2B	N615K	M2	ND	ID, DD	↓ Popen, ↓current density, ↓expression	Platzer et al., 2017; Li et al., 2019
GluN2B	V620M	M2	ND	DD, ID, Hpt	↓ Deactivation decay time, ↓ expression, ↑ Popen, ↓ proton inhibition	Platzer et al., 2017; Li et al., 2019
GluN2B	S628F	M2-M3	ND	ID, DD, Epi	Glu insensitivity, ↓ current density, ↓ expression	Platzer et al., 2017; Vyklicky et al., 2018
GluN2B	G689C	LBD-S2	LOF	DD, ID, Hpt	↓ Glu potency, ↓ expression, ↓ proton inhibition	Kellner et al., 2021
GluN2B	R696H	LBD-S2	GOF	Epi, ID, ASD	↑ Glu potency, ↑ deactivation decay time, ↓ current density, ↑ charge transfer, ↓ expression	Swanger et al., 2016; Platzer et al., 2017
GluN2B	S1415L	CTD	ND	ASD	↓ Expression, ↓ spine density, impaired PSD-95 and SAP-102 binding	Liu et al., 2017

LBD, ligand-binding domain (LBD-S1, LBD-S2); transmembrane domains, M1–M4; linker regions, S1–M2, M2–M3; CTD, carboxy-terminal domain; ATD, amino-terminal domain; DD, developmental disorder; ID, intellectual disorder; Epi, epilepsy/seizures; IS, infantile spasms; ASD, autism spectrum disorder; CVI, cortical visual impairment; ADHD, attention deficit hyperactivity disorder; Hpt, hypotonia; LGS, Lennox-Gastaut syndrome; LKS, Landau-Kleffner syndrome; LOF, loss-of-function; GOF, gain-of-function; ND, not determined; ↑, increased; ↓, decreased; Glu, Glutamate; Gly, glycine; Zn, zinc; Mg, magnesium; Popen, probability of opening; WT, wild type. *Surface expression quantified primarily in HEK293T cells.

A significant number of variants identified within the GluN subunits have been classified as *de novo*, a mutation that is observed first in offspring that is not present in the parents (XiangWei et al., 2018; Li et al., 2019). *De novo* mutations are on average more deleterious and have been established to be a significant cause of neurodevelopmental disorders including autism, epilepsy, and other intellectual disorders (Veltman and Brunner, 2012; Acuna-Hidalgo et al., 2016). It should also be noted that patients with *GRIN* variants are often heterozygous for mutations within the affected gene, and many are classified as dominant-negative, loss-of-function mutations that reduce NMDAR function. Other variants may lead to a gain-of-function of NMDARs in which the channel gating properties are altered (XiangWei et al., 2018). The dominant effects of both of these genetic variances interfere with the function of the wild type subunit and are therefore more detrimental in disease states. Single-point mutations can exacerbate the inefficiency of protein folding. However, if subunits remain stable enough to conform to their natively folded state, they will assemble with other subunits to form receptors (Gershenson, 2014; McEntagart et al., 2016). Further investigation into how these mutants alter intermolecular interactions involved in the folding, assembly, degradation, and trafficking will elucidate how cells accommodate DAVs within NMDARs and other ligand-gated ion channels.

Ogden et al. investigated the effects of having identical mutations within both GluN2A subunits of NMDARs compared to receptors containing only a single variant subunit. Specifically, the proline residues at position 552 were mutated to arginine residues. Receptors containing one mutant GluN2A_P552R displayed increased glutamate and glycine potency, a prolonged glutamate response time course, and increased charge transfer. No changes were seen in receptor surface expression or response amplitude. Additionally, transfection into neurons resulted in increased cation flux that caused dendritic blebbing and excitotoxic death. Meanwhile, receptors containing two GluN2A_P552R subunits showed decreased single-channel conductance and prolonged mean open times that were not observed in receptors with a single mutant subunit. This indicates that having two mutant subunits present in receptors can have additive adverse effects on receptor function, but only one mutant subunit is required for channel dysregulation. The patient identified in the study exhibited delayed psychomotor development, epilepsy, and intellectual disability. However, having only one copy of the *GRIN2A* gene containing a variant indicates that their NMDARs could contain 0, 1, or 2 mutant GluN2A subunits. Therefore, no direct correlations between the number of mutant receptors and the severity of clinical phenotypes can be made (Ogden et al., 2017).

**FIGURE 2:**
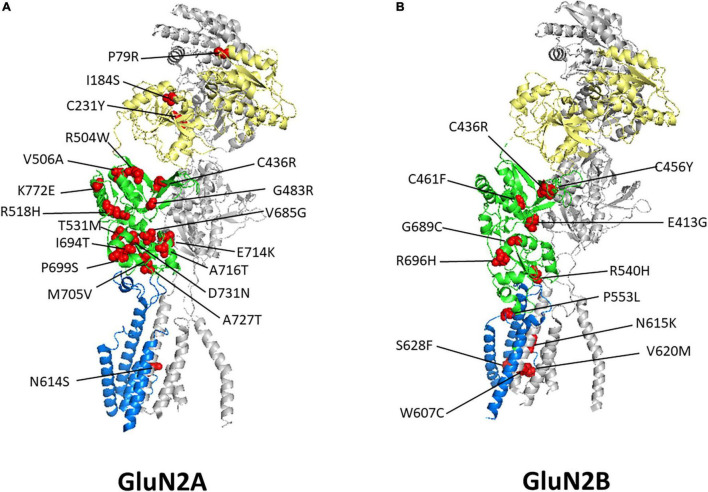
Disease-associated variant locations within GluN2 Subunits. Shown are heterodimer structures of half of an NMDAR containing one GluN1 subunit, in gray, and one GluN2A subunit **(A)** or one GluN2B subunit **(B)**, with the ATD in yellow, LBD in green, and TMD and linkers in blue. Disease-associated variants with reduced surface expression are shown as spheres and highlighted in red. GluN2A PDB: 7EU7 (Zhang Y. et al., 2021) GluN2B PDB: 6WHS (Chou et al., 2020). See Table 1 for details regarding these variants clinical phenotypes and functional consequences.

*N*-methyl-D-aspartate receptor variants that were shown to have reduced surface expression were often affected in their LBDs. Figure 2 displays the selected mutants from Table 1 in a 3D structure of human GluN1_GluN2A (Figure 2A; Zhang Y. et al., 2021) and the rat GluN1_GluN2B (Figure 2B; Chou et al., 2020) heterotetrameric receptors generated from their cryo-electron microscopy model. Within the ER, ligand binding may serve as a quality control checkpoint to demonstrate the functionality of receptors as suggested for the GluK2 subunit of kainate receptors (Mah et al., 2005).

The exact mechanisms that contribute to reduced surface expression have not yet been described in detail. Single-point mutations are enough to perturb the ability of a protein to adopt its correct structure and ability to oligomerize, leading to the accumulation of insoluble aggregates (Gershenson et al., 2014). In such cases, proteins are retained in the early secretory pathway and targeted for degradation. Additionally, disease phenotypes may arise due to improperly folded, assembled or immature oligomers being trafficked to the plasma membrane. This could result in increased receptor internalization and degradation, thereby fewer receptors displayed on the cell surface. To date, a majority of therapeutics targeting NMDARs are known only to act on receptors that are displayed on the surface of the cell. Variants that reduce the surface trafficking of the receptor make these treatments less efficacious. While the effects of these variants on channel kinetics, pharmacological properties, channel gating, ion permeability, and agonist potency have been investigated, little has been done to examine the molecular mechanisms of the proteostasis maintenance of these channels (Vyklicky et al., 2014, 2018; Hansen et al., 2021).

### The endoplasmic reticulum remains the critical factory for folding and assembly of newly synthesized *N*-methyl-D-aspartate receptors and other membrane proteins

Nearly one-third of proteins encoded by the mammalian genome are targeted to the ER for biogenesis. Membrane proteins are large proteins that inefficiently fold due to their complex structures and folding kinetics (Hebert and Molinari, 2007). For receptors to gain activity to perform their physiological role, they must fold and assemble correctly into tertiary and quaternary structures, allowing them to be trafficked to the plasma membrane (Balchin et al., 2016). Nonetheless, there remains a large knowledge gap in understanding the molecular details of the early biogenesis of many plasma membrane proteins. Investigation of targeted clinical therapies requires an understanding of the molecular mechanisms that underlie the processing of these proteins within the ER. For example, recent studies have found that the ER cargo protein ER-Golgi intermediate compartment protein-53 [ERGIC-53 (LMAN1)] plays a role in the trafficking of Cys-loop receptors within the CNS, but its role in glutamate receptor trafficking was not investigated (Fu et al., 2019).

Protein quality control mechanisms ensure proteins fold correctly, homeostasis is maintained, and proteins can perform their physiological functions. Additionally, they reduce the aggregation of misfolded or unassembled proteins that could lead to proteotoxicity (Balchin et al., 2016). Particularly, the ER protein quality control is composed of three general processes to ensure the biogenesis of proteins: chaperone protein interactions, a carbohydrate modification system, and a thiol-dependent system (Adams et al., 2019).

Typically, nascent protein chains are targeted to the secretory pathway, *via* a signal sequence on their amino-terminal that targets them to the translocon machinery on the ER membrane (Rapoport et al., 2017). Newly synthesized proteins enter the ER and interact with various chaperone proteins and folding enzymes. Together, these act to facilitate protein stability for folding, assembly, and maturation (Halperin et al., 2014). Additionally, these ER-resident proteins serve as retention signals for their substrates while they are bound and play a role in protein quality evaluation. Classical chaperones within the ER belong to the family of heat shock proteins (HSPs), including HSP40s, HSP70 [BiP (GRP78)], and HSP90 (GRP94). They function by binding promiscuously to hydrophobic regions of their substrates that contain alternating aliphatic residues that would normally be buried in the mature proteins (Behnke et al., 2016). BiP is the most abundant heat shock protein in eukaryotes, whose affinity for its substrates is regulated by ATP binding; such that when ATP is hydrolyzed, the substrate affinity increases (Hendershot, 2004). BiP has extensive roles within the ER and assists in protein folding, translocation, ER retention, regulation of the unfolded protein response, and promotion of ER-associated degradation (ERAD) (Pobre et al., 2019).

Secondly, the lectin proteins calnexin and calreticulin regulate the carbohydrate-dependent protein quality control (Wang et al., 2012; Kozlov and Gehring, 2020). As a majority of proteins enter the ER through the translocon, they are N-glycosylated by an oligosaccharyltransferase. The N-linked glycans, Glc_3_Man_9_GlcNAc_2_, are preassembled in the ER membrane and attach to proteins at multiple asparagine residues within the consensus sequence Asn-X-Ser/Thr, where X is any residue but proline (Wang et al., 2012; Breitling and Aebi, 2013; Lamriben et al., 2016). The glycan enhances protein solubility and serves as a reporter of the progression to the folded state. The glycan undergoes sequential removal of its sugars by a glycosidase, thereby allowing the substrate’s binding with calnexin and calreticulin, which tether the substrate and various folding cofactors to assist in the folding of the nascent protein (Hammond et al., 1994). After the final glucose is trimmed, the natively-folded protein continues through the secretory pathway from the ER to Golgi. Moreover, UDP-glucose glycoprotein glucosyltransferase 1 (UGGT1) recognizes non-natively folded proteins and re-engages the folding process by adding back a glucose molecule to the substrate (Sousa and Parodi, 1995; Adams et al., 2020). As an additional safeguard mechanism, especially during ER stress, di-glucosylated glycans can be recognized by malectin, which retains the protein in the ER to acquire prolonged assistance (Galli et al., 2011). Generating correctly folded proteins requires many cellular resources, so mechanisms to attempt recycling and proper folding are beneficial for cell survival.

The final primary quality control mechanism relies on a thiol-dependent system. The ER lumen is a highly oxidative environment that ensures the formation of disulfide bonds between cysteine residues (Hwang et al., 1992; Tu and Weissman, 2004). Protein disulfide isomerases are found abundantly in the ER and can aid in the formation, reduction, and/or isomerization of disulfide bonds to ensure native folding (Hatahet and Ruddock, 2007). They primarily facilitate the formation of disulfide bonds between proximal cysteine residues, as reactive thiols exposed on the protein can aggravate misfolding (Okumura et al., 2015; Fra et al., 2017). For example, Erp57/52 binds to exposed cysteine residues and catalyzes the formation of intra- or inter-molecular disulfide bonds (Frickel et al., 2004; Jessop et al., 2007). During each stage of these quality control pathways, major markers for proteins in non-native states can be generated. These include unprocessed N-glycans, exposed hydrophobic regions, and exposed thiol groups (Määttänen et al., 2010). Together these quality control mechanisms ensure that only correctly folded proteins are trafficked out of the ER.

**FIGURE 3:**
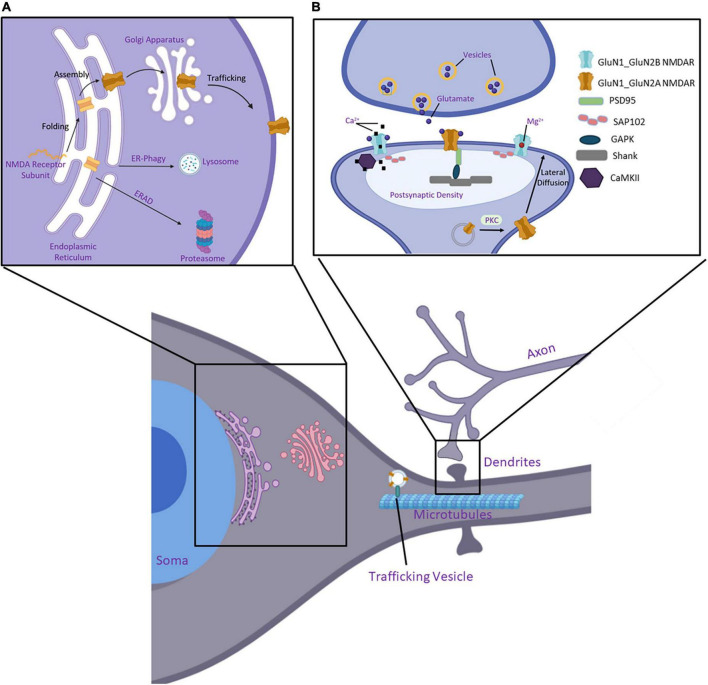
Essential components of the protein quality control pathway of NMDARs. **(A)** Nascent polypeptide chains of a GluN subunit enter the ER where chaperone proteins and folding enzymes assist its folding and assembly. Furthermore, it is trafficked to the Golgi apparatus, where it undergoes post-translational modifications, and eventually inserts into the plasma membrane to perform its function. If incorrectly folded within the ER, the protein is targeted to a degradation pathway, either through ER-phagy or ER-associated degradation (ERAD). **(B)** NMDARs are organized at synapses within the postsynaptic density (PSD). Protein kinase C (PKC) facilitates vesicle fusion with the membrane and the receptors laterally diffuse to the synaptic space. NMDARs are tethered to the PSD through interactions of their CTD with SAP102 and PSD95. These are further linked to Shank and Homer within the PSD. CaMKII binds and modulates NMDARs activity and calcium signaling at the synapse. Created with BioRender.com.

The biogenesis of NMDARs begins with the transcription of subunit *GRIN* genes and continues with the folding and assembly of receptors within the ER (Figure 3A). GluN1 subunits are expressed and translated in excess of GluN2 and GluN3 subunits (Chazot and Stephenson, 1997a; Huh and Wenthold, 1999). This ensures there is a reserve pool to oligomerize with newly synthesized GluN2 or GluN3 subunits as directed by signaling at the synapse, as GluN1 subunits are retained in the ER unless assembled to form tetrameric NMDARs (Okabe et al., 1999; Papadakis et al., 2004). Unassembled monomers of GluN2 subunits are also present in the ER, though to a lesser extent (Horak et al., 2008b; Kaniakova et al., 2012).

Several experimental models describe the proposed assembly of functional NMDARs. Numerous studies have provided evidence that homodimers of GluN1-GluN1 and GluN2-GluN2 first form, followed by their dimerization to form a heterotetrameric receptor (Meddows et al., 2001; Schorge and Colquhoun, 2003; Papadakis et al., 2004; Qiu et al., 2005; Hansen et al., 2010). A similar model has been proposed in which GluN1-GluN1 homodimers first assemble and act as a substrate in the formation of the receptor (Atlason et al., 2007). Another study determined that weak interdomain interactions occur between the ATDs of GluN1 subunits, forming a weak interaction after translation. These dimers dissociate and promote interaction with the GluN2 subunits as they are expressed, resulting in stabilized heterotetrameric NMDARs (Farina et al., 2011). On the contrary, Schüler et al. (2008) found that GluN1-GluN2 heterodimers are required for the formation of triheterotetrameric receptors. There is no conclusive evidence to evaluate these proposed models for NMDAR dimerization and future investigations should seek to delineate the assembly of functional NMDARs further. The formation of triheteromeric NMDARs raises the question of whether oligomerization is stochastic in the presence of multiple GluN2/GluN3 subunits or whether neuronal signaling or other cellular signaling processes that can alter the metabolic state of the cell, gene expression, and patterns of RNA splicing regulate assembly. It may be true that all observed instances occur at some stage of the assembly process that promotes specific subunit selection.

It should, however, be noted that iGluRs form intertwined tetramers arranged in a 1-2-1-2 pattern, as first observed by Sobolevsky et al. in their crystal structure of the GluA2 homotetrameric AMPAR and by other full-length iGluRs since (Karakas and Furukawa, 2014; Karczewski et al., 2020). It is proposed that ATD dimer interactions in GluA2 AMPAR subunits initiate dimer formation, and the formation of tetrameric arrangements occurs *via* interaction of the LBDs followed by pore formation in the TMDs (Greger et al., 2007). For a comprehensive review regarding the assembly of AMPARs see: Gan et al. (2015) and Schwenk et al. (2019).

Specific domains have established roles in the formation of heterotetramers. Like AMPARs, the ATD of NMDARs has been suggested to initiate dimer formation (Meddows et al., 2001; Papadakis et al., 2004; Hansen et al., 2010). Furthermore, the CTD of many membrane protein subunits contains ER retention motifs that keep unassembled and improper oligomers from exiting the ER (Jackson et al., 1990). These signals must be masked for receptors to be trafficked further in the secretory pathway. This can occur through oligomerization that masks the signal, phosphorylation of key residues, or interactions with trafficking factors, such as PDZ domain-containing proteins (Xia et al., 2001).

To further investigate the role of specific regions of GluN subunits in receptor assembly, Cao and others created truncated and chimeric cDNA constructs. Utilizing fluorescence resonance energy transfer (FRET) and co-immunoprecipitation (co-IP) techniques, they determined that the TMD of the subunits, but not the N-terminus, was necessary for the formation of both homodimers and heterotetramers (Cao et al., 2011). Remarkably, the reliance upon the TMD for assembly has also been observed for AMPARs (Gan et al., 2016). The extent to which the early secretory pathways overlap in the folding and trafficking of the different iGluR subfamilies is not well-understood in full. However, some papers suggest much deviation in their interaction partners, especially those of AMPARs and NMDARs, such that they are unique to their receptor type (Allison et al., 1998; Hansen et al., 2021).

The interactome of NMDARs within the ER remains unclear. It is hypothesized that NMDARs use common machinery within the ER for initial folding and assembly but specialized accessory proteins may facilitate as receptors mature. Nevertheless, proteins that interact with NMDARs within the ER to assist in the assembly and export of the receptors have not yet been elucidated. In the case of AMPARs, interactions with the proteins FRRS1L and CPT1c have been found to occur within the ER but dissociate before they reach the plasma membrane. It is thought that these proteins influence the assembly of AMPARs (Brechet et al., 2017). Moreover, the chaperone protein BiP has been found to coimmunoprecipitate with AMPARs, indicating they also utilize common machinery within the ER (Rubio and Wenthold, 1999). BiP has also been observed to interact with the GluN2A subunit in co-IP studies. However, this same study failed to detect an interaction between GluN2B and BiP (Zhang et al., 2015). This may indicate that the interaction with GluN2B is weak or unstable in comparison to the GluN2A subunit. Otherwise, this hints that while their sequences are quite similar, they may have preferential molecular mechanisms within the proteostasis network that facilitate the trafficking of subunit-specific NMDARs. This may be induced by the change in the GluN2A/GluN2B ratio that occurs during development, synaptic transmission, and synaptic plasticity.

### Cellular events involved in the forward trafficking and membrane insertion of *N*-methyl-D-aspartate receptors

Retention of subunits within the ER for complex proteins is a common quality control mechanism to ensure defective and unassembled proteins are not further released (Ellgaard and Helenius, 2003). General mechanisms as detailed above have been shown to facilitate NMDAR folding in the ER. For instance, in-depth characteristics of NMDARs exiting the ER have been elucidated, including a variety of post-translational modifications (Standley and Baudry, 2000). In a study carried out by Lichnerova et al., two glycosylation sites within the GluN1 subunit, Asn 203 and Asn 368, were found to be essential in the forward trafficking of NMDARs. In order to determine this, they replaced the Asn residues of 12 potential glycosylation sites of the GluN1 subunit with Gln residues individually and monitored the surface delivery of NMDARs. In contrast, mutating the 7 potential glycosylation sites of GluN2A and GluN2B subunits individually did not influence the surface delivery of these receptors (Lichnerova et al., 2015). Interestingly, another post-translational modification, palmitoylation, has been shown to play a role in modulating NMDARs’ exit from the ER. Two cysteine clusters on the CTD of both GluN2A and GluN2B are palmitoylated. However, the effects of these palmitoylation events are contrasting. The first cysteine cluster, identified proximal to the membrane, enhances the surface expression of NMDARs when palmitoylated. The second cluster, present in the middle of the CTD, results in decreased expression of NMDARs due to accumulation within the Golgi apparatus (Hayashi et al., 2009). Furthermore, mutations to the membrane-proximal cysteine cluster resulted in reduced surface expression of NMDARs while mutations to the second cysteine cluster resulted in increased expression (Mattison et al., 2012). This post-translational modification allows modulation of NMDAR surface expression and assists in anchoring the CTD to the plasma membrane once the receptor is inserted into the synapse (Hubalkova et al., 2021).

Also GluN1 subunits are retained in the ER due to retention signals typically found on their C-terminal domains (Scott et al., 2001; Hong et al., 2015). These retention signals are thought to be masked, allowing for the forward trafficking of receptors otherwise ensuring that unassembled or defective proteins are not released from the ER (Horak et al., 2008b; Horak and Wenthold, 2009). Consistently, previous research has shown that GluN subunits are retained within the ER unless they are assembled into heterotetramers. In mice lacking GluN1 subunits, GluN2 subunits accumulated and were found to be sequestered in the ER (Fukaya et al., 2003). In GluN1 subunits containing a C1 cassette, the ER retention motifs KKK and RRR can be found (Standley et al., 2000; Scott et al., 2001; Horak and Wenthold, 2009). However, to date, there has been no ER retention motif determined within the GluN2A CTD; yet interestingly, Qiu et al identified around 100 residues within the A2 segment of the GluN2A ATD that were found to be essential in preventing the forward trafficking of GluN2A-containing NMDARs (Qiu et al., 2009). The GluN1a ATD masked this ER retention action to permit forward trafficking, although the mechanisms of such masking remain unclear. As this notable ER retention was not observed in the GluN2B A2 segment, 10 residues that are not conserved between these two sequences were chosen for GluN2A mutagenesis studies including I176Y, F186K, M200L, A213S, S225P, D234E, L238Y, E242V, F253Y, and K270S. However, mutagenesis of each of these residues did not result in increased surface expression of the receptor, thereby not eliminating the ER retention activity located within this A2 segment. These results indicate that each of these residues alone is not sufficient for retention. Nevertheless, they may still play a role in the ER retention function. The authors further split this A2 segment into three parts consisting of the following residues (Ile 151–Asp192), (Asn193–Leu238), and (Ile239–Asn282) and fused them into pDisplay-GFP to monitor their surface labeling. Interestingly this experiment found that there was no significant difference in the surface fluorescence between these chimeras. This suggests that specific residues may act as a conformational ER-retention signal or as a multiple site-dependent ER retention motif. However, this study highlighted that ER-quality control mechanisms can differ between GluN2A and GluN2B-containing receptors (Qiu et al., 2009). Additionally, no specific ER retention signal sequence has been determined for the GluN2B subunit, however, truncation of the CTD up to residue 1,070 resulted in increased surface expression (Hawkins et al., 2004). Similarly, a study showed that functional receptor complexes are formed in the absence of either the GluN1 or the GluN2B C-terminus (Horak et al., 2008b). These results indicate that the CTD may play a critical role in receptor retention.

Though the mechanism by which the assembled tetrameric complex overrides the ER retention of the individual subunits has been studied in only a limited number of cases, these results suggest that the NMDARs may be controlled by certain signals indicating that the assembly process is complete rather than by masking of specific retention signals. Additional residues have been identified within the subunits that facilitate the forward trafficking of receptors. For example, the HLFY motif on the end of TM4 in the GluN2B subunit, which is not present in GluN1 subunits, was found to likely be involved in the export of NMDARs (Hawkins et al., 2004). Replacing each of these key residues with alanine, or truncating the GluN2B before the TM4 domain was found to disturb the surface trafficking of functional receptors (Yang et al., 2007; Horak et al., 2008a). It is believed that this motif may play a role as a conformational signal, ensuring proper orientation of the receptor domains. The key amino acids Trp 636 and Tyr 647/Thr 648 within the GluN1 TM3 and Trp 635 and Ser 645/Tyr 646/Thr 647 within GluN2B TM3 have been shown to regulate their surface delivery. These residues ensure subunits are retained in the ER until correct conformation of the TM3 domains is achieved to continue the forward trafficking (Horak et al., 2008a; Kaniakova et al., 2012).

Further mechanisms have been demonstrated to facilitate proteins exiting the ER such as the interaction of glutamate receptors with scaffold proteins. Recent work has highlighted the importance of the intrinsically disordered CTD of these receptors for such interactions. These interactions occur with a variety of membrane-associated guanylate kinases (MAGUK) proteins such as postsynaptic density protein 95 (PSD-95) and synapse-associated protein 102 (SAP102) (Sans et al., 2003; Petralia et al., 2005). PSD-MAGUK recognizes divaline motifs and helps regulate the ER retention of NMDARs (Standley et al., 2000). Many AMPAR interacting proteins have been found to influence the ER export, especially the members of the TARP family (Milstein and Nicoll, 2009). The C-termini of TARPs have an unresolved ER-export signal motif that promotes the forward trafficking of AMPARs whose mechanism of action has been proposed to be to mask the ER retention motifs on GluA subunits (Bedoukian et al., 2008). The genetic deletion of TARP γ-2 in *stargazer* mice results in forward trafficking of immature AMPARs and induces the unfolded protein response (Tomita et al., 2003; Vandenberghe et al., 2005). Further, upon overexpression of TARP γ-2, AMPARs that were accumulating intracellularly were trafficked to the cell surface (Kessels et al., 2009).

Evidence shows that the CTD of iGluRs plays a role in the secretion of receptors from the ER *via* interaction with other proteins (Jacobi and von Engelhardt, 2018; Warnet et al., 2021). The interacting proteins that can facilitate iGluR ER export are considerably diversified due to alternative splicing of the receptor subunits. In NMDARs, the GluN1 subunit has eight isoforms that arise from the *GRIN1* mRNA alternative splicing of exons 5, 21, and 22 (Zukin and Bennett, 1995). GluN1-1A is the dominant isoform whose splicing is regulated by neuronal activity (Laurie and Seeburg, 1994; Mu et al., 2003). Recent works have identified a primate-specific GluN2A isoform. This short GluN2A isoform can coassemble with GluN1 subunits to form functional receptors and was found to compose nearly a third of GluN2A subunits in the human cortex (Warming et al., 2019). These isoforms increase the molecular diversity of the channel and allow for diversified pharmacology and protein interaction partners that facilitate the delivery of these receptors. For a comprehensive overview of human peculiarities of iGluR splicing and editing see (Herbrechter et al., 2021).

Secondly, ligand binding may be a quality control mechanism for ER export of NMDARs that ensures receptors can undergo native conformational changes. She et al. (2012) showed glutamate but not glycine binding was required for the release of receptors from the ER; further, it required binding at both GluN2B subunits, not just one. This study indicated that there is a strong correlation between glutamate affinity and forward trafficking of GluN2B-containing NMDARs. Further, glutamate concentration within the ER has been demonstrated to be within millimolar ranges, which is sufficient for binding to NMDARs (Laurie and Seeburg, 1994). However, it should be noted that subunits with lower affinity binding sites and variants that impair ligand binding are still trafficked to the cell surface to some degree, but an apparent correlation between glutamate affinity and rate of receptor release was nonetheless observed. Additionally, ligand binding has been proposed as a gating quality control of AMPARs before ER secretion (Greger et al., 2002; Penn et al., 2008). Also, glycine binding has been demonstrated to play a role in the forward trafficking of NMDARs as demonstrated in a mutagenesis study of the ligand-binding site of the GluN1 receptor. Kenny et al. (2009) found that the point mutation D732A within the glycine binding site of GluN1 reduced the surface trafficking of NMDARs by 90%. Such redundant quality control mechanisms can ensure only mature and functional receptors are secreted. Whether these mechanisms occur constitutively during the biogenesis process, under specific circumstances, or for subunit specification remains to be investigated.

The forward trafficking from the Golgi apparatus to the cell surface and the postsynaptic organization of NMDARs has been elucidated in greater detail in the literature (Lau and Zukin, 2007; Petralia et al., 2009; Horak et al., 2014; Pérez-Otaño et al., 2016; Lira et al., 2020). NMDARs can be targeted to the plasma membrane through a canonical or non-canonical pathway. In the canonical pathway, glutamate receptors follow the secretory pathway from the soma of the neurons (Figure 3). Here the proteins exit the ER and enter the ER-Golgi intermediate compartment (ERGIC). Once in the Golgi, the receptors can be inserted into vesicles for trafficking to the plasma membrane, or vesicles are transported on the microtubular cytoskeleton and are delivered to dendrites (Viotti, 2016). In the non-canonical pathway, NMDARs interact with SAP97 and CASK, bypassing the ERGIC forward trafficking, and travel in vesicles to Golgi outposts (Wenthold et al., 2003; Jeyifous et al., 2009; Lin et al., 2013). It should also be noted that organelles involved in the secretory pathway are organized in neurons both within the soma and extend into dendritic spines. This allows for local protein synthesis of receptors very near their site of action. Giandomenico et al. (2022) provide an excellent review disusing proteostatic regulation within neurons. While the differences in proteostasis networks between somatic and dendritic ERs have not been elucidated in full, AMPAR subunits show distinct receptor formation and trafficking patterns. As demonstrated in hippocampal neurons, GluA1 is trafficked from the ER *via* the canonical pathway from the somatic ER (Jeyifous et al., 2009). However, secretion of GluA1 from dendritic ER has also been observed *via* interaction with SAP97. On the other hand, GluA2 accumulation in hippocampal neurons was observed in internal membranes along the dendrite (Greger et al., 2002; Perestenko and Henley, 2003).

Receptors are inserted into the membrane *via* exocytosis, which can occur directly at the synapse or in the extrasynaptic membrane. Recent studies have shown that DISC1 plays an essential role in the motility of NMDARs within the membrane. This process, known as lateral diffusion, occurs once receptors are inserted into the membrane where they can then move through the membrane into the synaptic cleft. Most commonly found NMDARs in extrasynaptic spaces are GluN2B-containing receptors. This study found that GluN2B subunits form complexes with TRAK1, which is a DISC1-associated trafficking factor. Additionally, this study shows that DISC1 interacts with the GluN1 subunit to regulate NMDARs in dendrites in mouse cortical neurons (Malavasi et al., 2018). An excellent review summarizes in great detail the trafficking of NMDARs from the ER to the synapse as presented by Horak et al. (2014). Briefly, once inserted into the plasma membrane, NMDARs can be located extrasynaptically or in the spine of dendrites within the postsynaptic density (PSD) (Figure 3B). The PSD serves to structurally organize synapses and mediate interactions between scaffold or adaptor proteins with receptors on the spine surface (Gao et al., 2013). SAP102 and PSD-95 can anchor NMDARs in the PSD *via* their interactions with Shank and Homer (Petralia et al., 2005). SAP102 shows a higher affinity with GluN2B subunits and PSD-95 has a higher affinity for GluN2A subunits (Sans et al., 2000; van Zundert et al., 2004). However, both scaffolding proteins have been found to interact with GluN2A and GluN2B within the PSD (Sans et al., 2003; Elias et al., 2008; Standley et al., 2012).

Postsynaptic Ca^2 +^ concentration increases as a result of NMDAR signaling, activating many kinases as part of its second messenger activity including Ca^2+^/calmodulin-dependent protein kinase II (CaMKII), protein kinase A (PKA), and mitogen-activated protein kinase (MAPK) (Roberson and Sweatt, 1996; Gardoni et al., 1998; Leonard et al., 1999; Wang et al., 2007). CaMKII can modulate signaling occurring through GluN2B-containing receptors and facilitates long-term potentiation (Zhou et al., 2007). PKC within the dendrites enhances NMDAR phosphorylation resulting in enhanced opening of the channel. Additionally, PKC and PKA facilitate NMDAR trafficking by promoting exit from the ER. PKC has additional action within the dendrites to facilitate the insertion of NMDARs into the plasma membrane (Lan et al., 2001; Scott et al., 2003).

### Clearance of unfolded proteins is an indispensable component of *N*-methyl-D-aspartate receptor homeostasis

Protein folding is an inherently error-prone process that does not always form functional proteins. Newly synthesized proteins are prone to misfolding and forming toxic aggregates within the crowded ER lumen (Ellgaard and Helenius, 2003; Hartl and Hayer-Hartl, 2009). Mutations may further aggravate the formation of insoluble aggregates resulting in proteotoxicity (Gershenson et al., 2014). If aggregates are not cleared, they may stay bound thereby sequestering BiP and other chaperone proteins within the ER and impeding further protein folding in the cell (Sörgjerd et al., 2006). Three main pathways constitute the protein quality control process to handle misfolded and aggregated proteins: the unfolded protein response (UPR), ER-associated degradation (ERAD), and ER-phagy (Kaufman, 2002; Smith et al., 2011; Lipatova and Segev, 2015).

First, the UPR is activated to restore protein homeostasis under ER stress conditions, when the ability of the ER to fold proteins is at capacity (Hetz et al., 2020). Three ER transmembrane protein sensors can initiate signaling cascades in response to unfolded proteins that result in the activation of the UPR pathway: inositol-requiring protein 1 (IRE1), protein kinase RNA-like ER kinase (PERK), and activation transcription factor 6 (ATF6) (Kaufman, 2002). Under physiological conditions, BiP interacts with both IRE1 and PERK and prevents their phosphorylation (Carrara et al., 2015; Kopp et al., 2018). BiP also keeps ATF6 inactive by preventing its translocation to the Golgi (Shen et al., 2002). Upon ER stress, BiP detaches from these sensors to interact with the accumulating protein within the ER lumen. IRE1 is activated by a self-transphosphorylation event bestowing its RNase activity (Shamu and Walter, 1996; Zhou et al., 2006). IRE1 removes an intron from X-box binding protein 1 (XBP1), shifting the open reading frame (Sidrauski and Walter, 1997; Yoshida et al., 2001). This results in the expression of a spliced XBP1 transcription factor that is known to upregulate genes involved in protein folding, assembly, secretion, and degradation (Kaufman, 2002; Shaffer et al., 2004). PERK is also activated by self-transphosphorylation. After activation, it phosphorylates eukaryotic translation initiation factor 2 subunit alpha (eIF2α), which leads to the inhibition of protein synthesis to reduce the folding load of the ER. The eIF2α also leads to the selective translation of ATF4, which promotes the translation of stress-related proteins like those involved in autophagy and ER folding (Teske et al., 2011). After the dissociation of BiP from ATF6 as a result of ER stress, ATF6 translocates to the Golgi (Sommer and Jarosch, 2002). Here it is cleaved by proteases resulting in a fragment, ATF6p50, with a bZIP nuclear transcription activation domain that allows it to act as a transcription factor (Haze et al., 1999). ATF6p50 translocates to the nucleus and regulates survival-related genes in conjunction with spliced XBP1 (Bommiasamy et al., 2009). ER proteostasis has recently been shown to be enhanced by proteostasis regulators that preferentially activate the ATF6 arm of the UPR. Interestingly, Wang et al. demonstrate that the proteostasis regulators AA147 and AA263 increased the surface expression and thereby assembly and trafficking of variant γ-aminobutyric acid type A (GABA_A_) receptors implicated in genetic epilepsies. This study further identified that these proteostasis regulators increase the protein levels of ER chaperone BiP as well as lectin mannose-binding 1 (LMAN1), which facilitates the secretion of proteins from the ER to the Golgi (Wang M. et al., 2022). Together these branches of the UPR pathway act to attenuate protein expression, degrade misfolded protein, and upregulate the expression of folding proteins within the ER. The role of the UPR on the folding and degradation of NMDARs needs to be explored, as it presents a novel therapeutic target to ameliorate variant NMDAR trafficking.

Endoplasmic reticulum -associated degradation is the second pathway in which cells attempt to reestablish homeostasis. Here, misfolded proteins are targeted to the proteasome for degradation. Misfolded proteins within the ER are recognized by markers for improper folding as described in prior sections. Once misfolded proteins are recognized, they are retro-translocated out of the ER and into the cytosol (Nakatsukasa and Brodsky, 2008). Membrane-associated E3 ubiquitin ligases recruit E2 ubiquitin-conjugating enzymes and facilitate the transfer of the ubiquitin from the E2 to the misfolded protein substrate (Oh et al., 2018). The ubiquitinated protein is then removed from the membrane by valosin-containing protein (VCP/p97) in an ATP-dependent fashion and brought to the proteasome for degradation (Ye et al., 2001). Different E3 ligases are essential in the ubiquitination of misfolded proteins and are dependent on the location of the misfolding. Proteins containing misfolded domains within the lumen or membrane regions are targeted to the HRD1 complex (Bordallo et al., 1998), designated as ERAD-L and ERAD-M, respectively. Misfolded domains within the cytosolic compartment are targeted to the Doa10 (Swanson et al., 2001) complex for degradation, which is known as ERAD-C (Carvalho et al., 2006). It is estimated that 12–15% of cellular proteins are eliminated *via* ubiquitination (Duttler et al., 2013) The ERAD pathway ensures that the buildup of misfolded proteins is mitigated to reduce the proteotoxic effects of accumulating aggregates. A very limited number of E3 ligases have been studied in the context of NMDARs. For example, Nedd4 was shown to interact with GluN2D subunits and positively regulate their ubiquitin-dependent degradation (Gautam et al., 2013). In addition, F-box only protein 2 (Fbxo2), a substrate recognition part of the SCF (SKP1-CUL1-F-box protein) E3 ligase complex, interacts with high-mannose glycans of GluN1 subunits and positively regulates their ubiquitination (Kato et al., 2005). Interestingly, in *Fbox2* knockout mice, the surface expression of GluN1 and GluN2A, but not GluN2B, was increased (Atkin et al., 2015).

Thirdly, large bulk proteins and aggregates can be targeted for lysosomal degradation *via* ER-phagy, which is a selective form of autophagy that protects the cells from excessive ER stress (Mochida et al., 2015; Chino and Mizushima, 2020). ER-phagy receptors, such as FAM134B and SEC62, reside in specific structures of the ER; FAM134B is found in curved portions of the ER membrane while SEC62 is found in flat portions of the ER sheets (Khaminets et al., 2015; Fumagalli et al., 2016). These receptors respond to cell stress resulting from misfolded protein accumulation, starvation, and calcium imbalance within the ER lumen and link their respective ER domains to autophagy machinery (Grumati et al., 2018). Specifically, macro-ER-phagy involves the sequestration of ER fragments into autophagosomes that are then delivered to lysosomes for degradation (Lipatova and Segev, 2015). ER-phagy serves as an alternate clearance pathway for misfolded proteins and plays a role in both ER homeostasis and ER quality control. This pathway is especially important for large or unfoldable aggregates that cannot activate the ERAD pathway, cannot be transported through the translocon, or accumulate at the ER exit site. Such instances have been demonstrated for procollagen aggregates (Omari et al., 2018; Cunningham et al., 2019; Forrester et al., 2019; Fregno and Molinari, 2019). Since the GluN2B subunit has the LC3-interacting regions (LIRs) (Jacomin et al., 2016), the involvement of the lysosome in its degradation needs to be further investigated.

There are further peripheral quality control mechanisms present in the Golgi complex and the plasma membrane that result in the internalization and degradation of misfolded membrane proteins that escaped ER quality control (Arvan et al., 2002; Okiyoneda et al., 2011; Babst, 2014; Hellerschmied et al., 2019). The fate of the cell is determined by the activity of each of these clearance pathways, for if the ER stress is prolonged or too severe, the cell will undergo apoptosis (Szegezdi et al., 2006).

It is unclear how DAVs of NMDARs influence the proteostasis network that regulates their folding, assembly, degradation, and trafficking. The limited knowledge comes from the study of DAVs of GABA_A_ receptors by carrying out quantitative interactome proteomics to identify and compare the interactomes for wild type and misfolding-prone GABA_A_ receptors carrying the A322D mutation in the α1 subunit (Wang Y.J. et al., 2022). 125 proteins were identified in the interactome for wild type GABA_A_ receptors, 105 proteins were identified in the interactome for the α1(A322D)-containing GABA_A_ receptors, and 54 proteins overlap within the two interactomes, indicating that the mutation substantially influences the GABA_A_ receptors-interacting network. Further bioinformatics analysis showed that the mutant receptors preferentially interact with a subset of ERAD factors, such as VCP and an E3 ubiquitin-protein ligase TRIM21 (tripartite motif-containing protein 21). This is consistent with the result that the mutant is excessively disposed of by the ERAD pathway. Since the DAVs of an ion channel likely utilize a differentiating proteostasis network compared to wild type, potentially it is feasible to adapt such a network to selectively target the mutant for functional rescue.

### Understanding *N*-methyl-D-aspartate receptor variant-specific characteristics throughout the entire protein quality control process may provide valuable knowledge for precise and targeted pharmacological therapies of *N*-methyl-D-aspartate receptors

**FIGURE 4:**
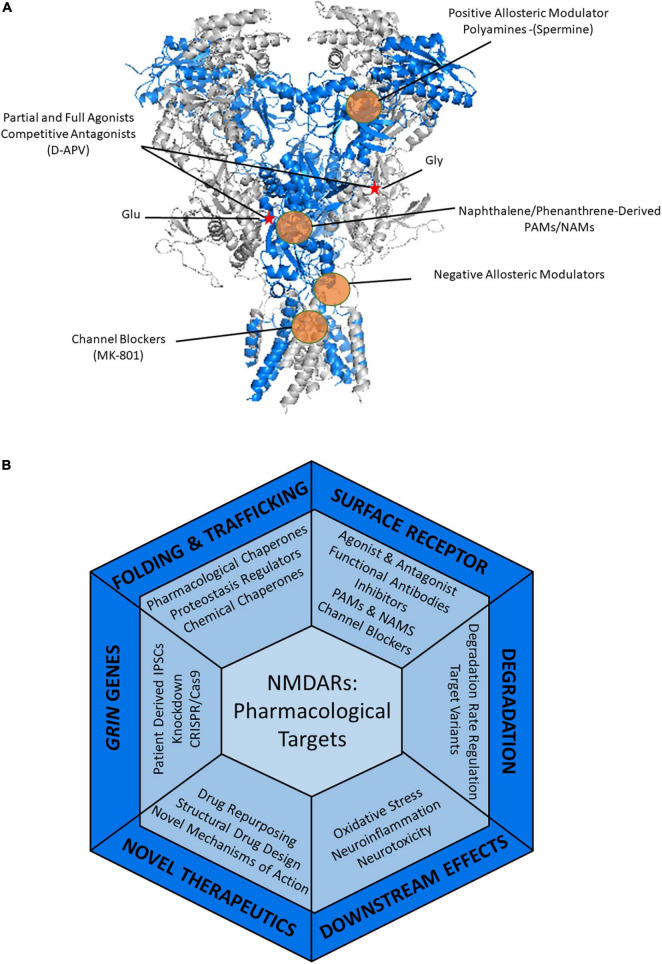
Therapeutic strategies to treat NMDAR-related diseases. **(A)** Pharmacology of NMDARs. Heterotetrameric structure of rat GluN1_GluN2B (PDB: 4PE5) with the GluN1 in gray and the GluN2B shown in blue. Glutamate and glycine binding sites are indicated by a red star. Examples of known modulatory sites of different pharmacological agents targeting NMDARs are highlighted by the orange circles. GluN2B selective molecules, such as ifenprodil and polyamines including spermine, bind in the ATD of NMDARs. Agonists and antagonists of NMDARs bind within the LBD. Positive and negative allosteric modulators can act within the LBD and throughout the TMD regions. Channel blockers, such as memantine and MK-801 bind within the pore in the TMD, thus blocking the channel. **(B)** Current and potential pharmacological and therapeutic approaches to target the NMDARs containing disease-associated variants throughout the protein quality control pathway.

Since the discovery of NMDARs, understanding how to modulate receptor function with pharmacological agents has been of great interest (Watkins, 1981). Studies in the last decade have provided great detail into the structure (Karakas and Furukawa, 2014; Vyklicky et al., 2014) and gating kinetics of NMDARs (Murthy et al., 2012; Dolino et al., 2017; Amin et al., 2021a), which have allowed for a better understanding of how drugs interact with and modulate these receptors. Figure 4A shows the binding sites of common drugs targeting NMDARs. These include partial and full agonists that can bind orthosterically at the glycine and glutamate binding sites; competitive antagonists, such as D-APV, also share these binding sites (Davies et al., 1982; Davis et al., 1992). Channel blockers, such as MK-801 and ketamine, inhibit the movement of ions through the channel, thus diminishing the current (Zorumski et al., 2016; Song et al., 2018). These drugs have been valuable for researchers to better understand the physiology of these receptors. Of particular interest, ketamine is effective in the treatment of major depressive disorder, possibly through its antagonistic effects on the receptor (Grady et al., 2017). However, many pharmaceutical therapeutics have limited clinical applications as NMDARs are crucial for normal synaptic transmission within the CNS. Particularly it has been found that full agonists of NMDARs have severe clinical side effects, such as off-target activity or excessive NMDAR activation or inhibition (Lipton, 2004; Hackos and Hanson, 2017). While some of these compounds are great for *in vitro* studies, the concentration required to have clinical effects results in undesirable side effects (Parsons et al., 1999).

Positive and negative allosteric modulators have become of greater interest in modulating the activity of NMDARs as they moderately regulate activity compared to more drastic effects caused by full agonists and antagonists (Mony et al., 2009; Hansen et al., 2018). Positive allosteric modulators (PAMs) have a potentiating effect on the receptors either by decreasing agonist EC50 (half-maximal effective concentration), increasing the maximal current, or both (Hackos and Hanson, 2017). PAMs are beneficial when the NMDARs of interest have hypofunction. Negative allosteric modulators (NAMs) reduce the response of the receptor to its ligands, either partially or fully (Burnell et al., 2019). NAMs would be beneficial for treating NMDAR hyperfunction. Hypofunction is the result of mutations that are deemed loss-of-function (LOF), in which the variant receptor lacks the molecular function of the wild type receptor (Lemke et al., 2016; Table 1). On the contrary, hyperfunction is typically the result of mutations that are deemed the result of gain-of-function (GOF), in which the variant receptor has a new or enhanced activity (Lemke et al., 2014).

Since the different NMDAR subunits display variation in their physiological properties as well as their anatomical expression, drugs that can target specific subunits are desirable not only for receptor modulation but for pharmacological therapies that seek to enhance the trafficking of variant NMDARs (Zhang and Luo, 2013; Sun et al., 2018). Novel approaches to target specific subunits of NMDARs can be achieved through structural-based drug design. Alternative structural-based approaches are demonstrated in the novel study in which, Tajima et al. generated an antibody specific for GluN2B-containing receptors that acts to inhibit the function of the channel by stabilizing the closed state of the channel (Tajima et al., 2022). The introduction of structural-based stabilizing antibodies into the CNS to promote the stability of variant receptors is an appealing future application. Many allosteric modulators have also been shown to have subunit specificity in their actions. Ifenprodil has inhibiting effects (Williams, 1993; Chenard and Menniti, 1999) and the polyamine spermine has potentiating effects (Traynelis et al., 1995) on NMDARs containing GluN2B subunits, while 24-s-HC (Paul et al., 2013) an endogenous neurosteroid, potentiates all receptor subtypes, though to different degrees (Hackos and Hanson, 2017; Burnell et al., 2019).

The varying pharmacological responses that can occur due to receptor subunit composition suggest that drug discovery may have to be tailored to these different receptor combinations (Temme et al., 2018). This is especially important in the case of triheteromeric receptors that are predominant in the CNS to avoid adverse effects (Rauner and Köhr, 2011; Tovar et al., 2013; Hansen et al., 2014; Stroebel et al., 2018). Positive modulation of a single specific subunit while also imparting an inhibitory effect on remaining subunits is another possibility that could have therapeutic benefits to variant NMDARs exhibiting hyperfunction. For example, the naphthalene-derived compound UBP684 potentiates receptors containing GluN2D subunits but has an inhibitory effect on all other subunits (Sapkota et al., 2017).

Previous studies have indicated that variant NMDARs have different degrees of responsiveness to drugs. Memantine, an NMDAR blocker used as a treatment for Alzheimer’s disease, has been investigated in GOF mutants within the GluN2B subunit (Briggs et al., 2016). Glutamate-induced currents were monitored for wild type and mutant receptors of GluN2B_N615I, both of which were effectively inhibited by memantine (Mullier et al., 2017). In contrast, the mutant GluN2B_V618G was resistant to memantine effects and showed no reduction in the current (Chen et al., 2020).

Restoring the surface expression and trafficking of these mutant receptors may be enough to bring NMDAR signaling into balance and restore physiological function. Restoration of surface trafficking has been demonstrated using pregnenolone sulfate, a PAM of receptors containing GluN2A or GluN2B subunits was found to increase the number of functional receptors on the cell surface within cortical neurons by 60–100% (Horak et al., 2004; Kostakis et al., 2013). However, many variants show changes in their agonist EC50s, which are further modulated by PAMs. Therefore, combination therapies may be an ideal treatment option, to first restore their surface expression and then modulate their activity. Further complications arise given that NMDARs are ubiquitously expressed within the CNS (Castillo et al., 2013; Mantuano et al., 2015). GluN2A localizes primarily to the synapse while GluN2B has significant roles synaptically and extrasynaptically (Rao and Craig, 1997; Rumbaugh and Vicini, 1999; Gardoni et al., 2006). Additionally, NMDARs can be found within the CNS in astrocytes, oligodendrocytes, and various glia (Murugan et al., 2011; Palygin et al., 2011; Gautier et al., 2015). Non-neuronal NMDARs have been detected in many tissues including the heart, pancreas, lung, kidney, and various other tissues (Inagaki et al., 1995; Deng et al., 2002; Deng and Thomson, 2009; Anaparti et al., 2015). Hogan-Cann and Anderson (2016) summarize their physiological roles in tissues outside of the CNS. This increases the likelihood that these drugs will have off-target effects. Additionally, for these therapies to target neuronal NMDARs, they must be able to bypass the blood-brain barrier (BBB) or be delivered directly into the CNS. NMDARs expressed on the surface of endothelial cells are found lining the BBB and modulate its permeability (Sharp et al., 2003; András et al., 2007). It was found that GluN3A containing NMDARs activity induces the Rho/ROCK pathway that increases the permeability of the BBB *via* phosphorylation of myosin (Mehra et al., 2020).

Treatment of DAVs must take into account whether they are GOF or LOF to modulate the receptor in the right direction. However, current drugs are only known to act on receptors displayed on the surface of the cell. What if these mutations result in reduced or ablated surface trafficking? What therapeutics can be used to restore the surface expression of these receptors? Figure 4B demonstrates broad approaches in which mutant-subunit containing NMDARs could be targeted to restore function or modulate their folding and trafficking.

Gene therapy may allow for the effective knockdown and replacement of mutant genes. Mice with a global LOF *GRIN1* allele showed deficits in cognitive behaviors, similar to those seen in *GRIN1* encephalopathies. To allow for selective expression, a neo cassette was inserted into intron 19 within the *GRIN1* gene and was flanked by *loxP* sites. *Cre* recombinase was then used to conditionally revert the locus to wild type through the knockdown of the LOF *GRIN1* allele. This rescued the mice and demonstrated that this LOF allele could be restored to wild type. Phenotypically, this rescue also resulted in improvements in cognitive function into adulthood (Mielnik et al., 2021). This study shows that recovery of neurological defects is possible, even into adulthood, demonstrating that therapeutic intervention in adult patients is a potential therapeutic target.

High-throughput screening (HTS) of the current list of Food and Drug Administration’s (FDA) approved drug library may uncover new mechanisms of action for drugs unknown to target NMDARs. Therapeutic benefits may also be uncovered through drug repurposing, such as seen by Tobramycin. Tobramycin is an FDA-approved drug for the treatment of several bacterial infections including septicemia, lower respiratory tract infections, and CNS infections.^1^ Tobramycin has also been found to display potentiating effects on GluN2B containing NMDARs and has restored the synaptic currents of mutant receptors (Masuko et al., 1999; Tang et al., 2020).

The methodologies that target specific biogenesis regulators and proteostasis processes such as folding, trafficking, and degradation require a better understanding of the molecular mechanisms that facilitate NMDARs in their maturation through the secretory pathway (Balch et al., 2008). Small molecules that are known chemical chaperones, pharmacological chaperones, or proteostasis regulators could be screened to determine if they have advantageous effects in modulating the folding and trafficking of variant NMDARs. Chemical chaperones, such as glycerol, dimethyl sulfoxide, and trehalose, among others, assist in correcting the misfolding, mislocation, and aggregation of mutant proteins associated with diseases. They have general effects on these processes and often exert effects on multiple proteins (Cortez and Sim, 2014). Pharmacological chaperones are small molecules that directly bind and stabilize proteins during their biogenesis and/or trafficking. Therefore, pharmacological chaperones are specific to their target proteins, serving as scaffolds to assist in protein folding and route target proteins correctly to the functional location (Wang et al., 2014; Liguori et al., 2020). Proteostasis regulators are an additional class of molecules that have effects that adjust proteostasis networks and increase the surface trafficking of proteins without making direct interaction with their target proteins (Mu et al., 2008; Plate et al., 2016).

While little is understood about the biogenesis and degradation pathways of NMDARs, many works have investigated these pathways for GABA_A_ receptors (Fu et al., 2016). GABA_A_ receptors are large, multi-subunit, ligand-gated neurotransmitter receptors that mediate a role in inhibitory neurotransmission and are known to inefficiently fold within the ER. Affinity purification mass spectrometry-based proteomics study identified the proteostasis network for GABA_A_ receptors, which regulates their folding, assembly, degradation, and trafficking (Wang et al., 2013; Wang Y.J. et al., 2022). Moreover, it was found that a Hsp90 in the ER lumen, Grp94, regulates the ERAD of these receptors by delivering them to HRD1 mediated ubiquitination pathway (Di et al., 2016). Further studies have sought to increase the surface expression of these receptors. It was found that the FDA-approved drugs dinoprost and dihydroergocristine enhanced the surface expression of mutant subunit-containing GABA_A_ receptors and restored the synaptic currents. These drugs were able to reduce the Grp94-mediated ERAD pathway and enhance the mutant receptors’ interaction with folding chaperones BiP and calnexin, thereby increasing the incorporation of mutant subunits into functional receptors (Di et al., 2021). Further, small molecule compounds have been used to target the proteostasis network. KM04794 has recently been shown to modulate the BiP chaperone system in the synthesis of insulin and enhance the efficiency of folding. KM04794 was found to accumulate in the ER and inhibit activation of the UPR thereby reducing protein aggregation and cell death (Miyake et al., 2022). While intracellular actions of many small molecules and allosteric modulators have not been described in literature, the differential response of variants should be investigated in future studies seeking to examine therapeutics targeting NMDAR proteostasis.

Pharmaceuticals may also have multiple effects that will benefit NMDAR DAVs dysregulation. For example, the small molecule AA147 is a proteostasis regulator that activates the ATF6 arm of the UPR, thereby reducing ER stress. In addition to this mechanism of action, AA147 also protects against glutamate-mediated excitotoxcity by activating nuclear factor erythroid 2- related factor 2 (NRF2) regulated oxidative stress response, and thus reduces oxidative damage to cells (Rosarda et al., 2021). While there is currently no direct evidence that NMDAR signaling results in the oxidative stress that this drug is capable of reducing, it raises an additional consideration for dual effects of future therapeutics. If modulating the number of NMDARs on the cell surface is to be considered, one must also consider that signaling may then become imbalanced and will need to be addressed. Additionally, targeting downstream effects resulting from increased signaling of NMDARs, such as neuroinflammation and neurotoxicity, will be of great benefit to patients, as it may prevent cell death and other adverse effects.

Together these approaches provide various future directions to understanding the regulation of NMDAR folding, assembly, degradation, and trafficking and provide novel therapeutic approaches for targeting DAVs. Much foundational work must still be done to investigate the underlying mechanisms of NMDAR proteostasis. However, these approaches could be applied to all iGluR family members in an attempt to ameliorate disease phenotypes associated with DAVs in these receptors.

## Discussion

In this review, we have summarized important protein quality control mechanisms within the biogenesis pathway of NMDARs. Further, we have summarized the effect of disease-associated variants on this pathway and discussed current and future approaches for the pharmacological targeting of NMDARs containing disease-associated variants. It is critical to functionally assess and elucidate the effects of such DAVs on receptor structure, gating, and impacts on receptor signaling, of which, much work has been done. However, little has been done to elucidate the effects of these variants in the early biogenesis process. It is important to get a complete picture of the effects of these variants, as reduced current could be caused by a several factors, and the disease phenotype alone is not enough to determine the impacts of the variant’s dysregulation on receptor function. This is highlighted by the fact that some DAVs exhibit similar phenotypes despite differentially enhancing or reducing the activity of receptors.

Further, it is of great importance to determine essential interaction partners of each NMDAR subunit that are involved in the folding, trafficking, and degradation. In order to target these receptors to ameliorate disease, they must first be properly trafficked to the cell surface. These pathways present promising therapeutic targets to regulate the surface expression of NMDARs. Subunit-specific expression of NMDARs is critical for development and due to varying physiological properties, GluN2A and GluN2B cannot be substituted for one another. It must be considered whether these subunits utilize different pathways, under basal ER processing or if specific events only occur in response to synaptic signaling. Further, what impacts DAVs have on the early biogenesis pathway must be elucidated.

Additionally, many experiments that investigate the physiological properties of disease-associated variants and the effects of pharmaceuticals have been carried out primarily in HEK293T cells and primary neuron cultures. While these studies have been crucial in discovering more about the effects of these mutants, animal models and patient-derived induced pluripotent stem cells will provide a greater understanding of the physiological context in which these DAVs result in their phenotypes. While these receptors are important to target for pharmaceutical intervention, it must also be considered that these therapeutics must be able to cross the blood-brain barrier to have their desired effects on neurodevelopmental and neurodegenerative diseases. Further, triheteromeric receptor composition complicates the efficacy of drug treatments on modulating NMDARs. Understanding how NMDARs are properly folded, assembled, and degraded may open up new pathways that can serve as pharmaceutical targets to restore receptor surface expression and thereby function in individuals harboring disease-associated variants.

## Author contributions

TMB wrote the original draft. All authors reviewed and edited the text and approved the submitted version.

## Conflict of interest

The authors declare that the research was conducted in the absence of any commercial or financial relationships that could be construed as a potential conflict of interest.

## Publisher’s Note

All claims expressed in this article are solely those of the authors and do not necessarily represent those of their affiliated organizations, or those of the publisher, the editors and the reviewers. Any product that may be evaluated in this article, or claim that may be made by its manufacturer, is not guaranteed or endorsed by the publisher.

## Footnotes

1
https://www.accessdata.fda.gov/scripts/cder/daf/index.cfm?event=overview.process&ApplNo=065122
